# Cerebral Small Vessel Disease: Early-Life Antecedents and Long-Term Implications for the Brain, Aging, Stroke, and Dementia

**DOI:** 10.1161/HYPERTENSIONAHA.122.19940

**Published:** 2023-09-21

**Authors:** Ellen V. Backhouse, James P. Boardman, Joanna M. Wardlaw

**Affiliations:** Centre for Clinical Brain Sciences (E.V.B., J.P.B., J.M.W.), University of Edinburgh, Scotland, United Kingdom.; MRC UK Dementia Research Institute (E.V.B., J.M.W.), University of Edinburgh, Scotland, United Kingdom.; MRC Centre for Reproductive Health (J.P.B.), University of Edinburgh, Scotland, United Kingdom.; Edinburgh Imaging (J.M.W.), University of Edinburgh, Scotland, United Kingdom.

**Keywords:** cognition, education, epidemiology, policy, socioeconomic factors

## Abstract

Cerebral small vessel disease is common in older adults and increases the risk of stroke, cognitive impairment, and dementia. While often attributed to midlife vascular risk factors such as hypertension, factors from earlier in life may contribute to later small vessel disease risk. In this review, we summarize current evidence for early-life effects on small vessel disease, stroke and dementia focusing on prenatal nutrition, and cognitive ability, education, and socioeconomic status in childhood. We discuss possible reasons for these associations, including differences in brain resilience and reserve, access to cognitive, social, and economic resources, and health behaviors, and we consider the extent to which these associations are independent of vascular risk factors. Although early-life factors, particularly education, are major risk factors for Alzheimer disease, they are less established in small vessel disease or vascular cognitive impairment. We discuss current knowledge, gaps in knowledge, targets for future research, clinical practice, and policy change.

Cerebral small vessel disease (SVD) refers to a syndrome of clinical and neuroimaging findings in the white and subcortical gray matter resulting from pathologies in the small perforating cerebral arterioles, capillaries, and venules, manifesting on computed tomography or magnetic resonance imaging or pathology examination as white matter hyperintensities (WMHs), small subcortical infarcts, lacunes, enlarged perivascular spaces, microbleeds, and atrophy.^1,2^ Clinically, SVD presents as ischemic and hemorrhagic stroke, gait and balance dysfunction, and behavioral and neuropsychiatric symptoms. It is the leading cause of vascular cognitive impairment (VCI), responsible for up to 45% of dementias either as vascular or mixed with Alzheimer disease^3^ (AD), affecting all major cognitive domains.^4^

SVD is often attributed to common vascular risk factors, particularly hypertension, smoking, and diabetes,^5^ but the proportion of variance of SVD lesion burden explained by these risk factors combined is small (≈2%),^6^ and risk factor modification clinical trials have so far had limited effects on preventing recurrent stroke, cognitive decline, and WMH progression (eg, the Secondary Prevention of Small Subcortical Strokes Trial [SPS3] trial^7^). Furthermore, although WMHs are considered a primary pathology in VCI^8^ with a dose-dependent effect on cognition, risk of cognitive decline in those with WMHs varies considerably,^9^ suggesting other factors contribute to SVD pathology and associated cognitive decline and dementia.

Growing evidence about protective factors for SVD, stroke, and dementia emphasizes a life-course model with a key role for early-life factors. The World Health Organization identified a life-course approach as a priority for policy action in the Health 2020 framework^10^ and in their intersectional global action plan for optimizing brain health across the life course.^11^ In 2020, reports by the World Health Organization–United Nations Children’s Fund (UNICEF) –Lancet Commission and the Lancet Commission on Dementia Prevention highlighted the lifelong, intergenerational benefits of investment in children’s health and early development including reducing preterm birth, poor nutrition and growth, prioritizing education for all, and reducing deprivation and inequality.^12,13^ The Lancet Commission advocated for an approach to dementia prevention focused on enhancing cognitive resilience in later life by building cognitive reserve earlier in life.^13^

Reserve explains individual differences in clinical status in relation to neuropathology.^14^ An individual may be able to sustain cognitive function despite SVD pathology due to a larger brain or synapse count (brain reserve), reduced brain pathology over time (brain maintenance), or more efficient brain networks that are less susceptible to disruption (cognitive reserve).^14^ Established determinants of reserve include early-life cognitive ability (IQ), education, and occupation, but other factors operational during childhood, which may impact reserve, include an adverse prenatal environment (eg, due to maternal smoking, preeclampsia, or fetal growth restriction), perinatal brain injury, suboptimal childhood nutrition, a heightened stress environment, and socioeconomic deprivation (Figure).^15,16^

**Figure.**
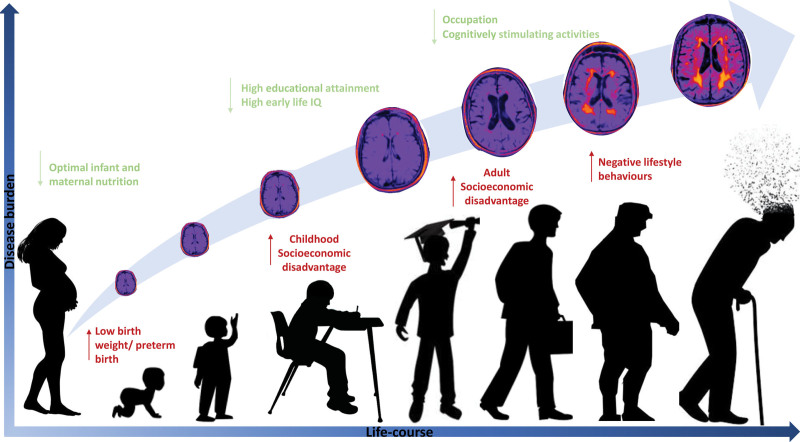
Protective and susceptibility factors for small vessel disease, stroke, and dementia.

In this review, we summarize current evidence for early-life effects on SVD, stroke, and dementia focusing on epidemiological studies of prenatal nutrition, early-life cognitive ability, education, and childhood socioeconomic status (SES).

## DEVELOPMENTAL ORIGINS OF HEALTH AND DISEASE

Following several landmark studies, Barker determined that the period from conception to birth, and the first few years of life, is critical in influencing disease susceptibility throughout life. According to the developmental origins of health and disease hypothesis, adverse intrauterine environmental exposures including stress and poor nutrition can induce permanent changes in fetal development, which, in combination with exposures during childhood and adulthood, increase vulnerability to chronic diseases in adulthood.^17^

Direct study of the intrauterine environment is challenging, but birthweight, standardized by gestational age and sex, reflects fetal growth, which is a reliable proxy of favorable or unfavorable conditions. Barker found people with lower birthweight, due to poor fetal growth rather than prematurity, had higher risk of cardiovascular disease and vascular risk factors in adulthood.^18^ Epidemiological studies have subsequently confirmed these findings, and relations between size at birth and disease in later life, particularly cardiovascular disease, are now well established.

Periods of famine, such as the Dutch famine of 1944 to 1945 and the Chinese great famine of 1959 to 1962, have revealed direct adverse effects of restricted prenatal nutrition on health outcomes including increased vascular risk factors and a higher prevalence of cardiovascular disease.^19^

## DEVELOPMENTAL ORIGINS OF HEALTH AND DISEASE AND CEREBROVASCULAR DISEASE

### Stroke

Despite ample evidence for the developmental origins of health and disease hypothesis in respect to cardiovascular disease and vascular risk factors, the potential role of early-life factors in CVD has received relatively little attention.

Large cohort studies report inverse associations between birth weight and stroke incidence and mortality^20–24^ with hazard ratios ranging from 0.48 to 0.84 for every 1-kg increase in birth weight (Table 1).^21,22,24^ Adjustment for confounders varies widely between studies, and none adjust for all relevant confounders likely to influence later CVD (eg, vascular risk factors, SES, education, and gestational age), meaning that the current findings may be inflated. The largest study (n>70 000) reported an 11% decrease in risk of nonfatal stroke for every 454-g increase in birth weight, independent of vascular risk factors.^20^

**Table 1.**
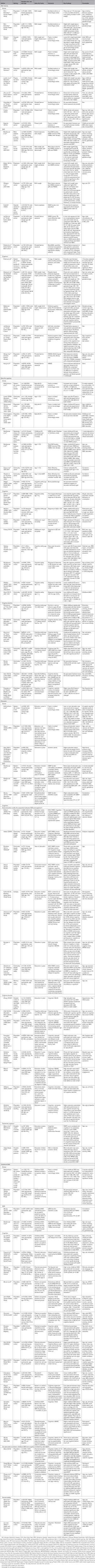
Overview of the Key Papers Examining Associations Between Birth Factors, Early-Life Cognition, Education, and Childhood SES and Later-Life Stroke, SVD, and Cognitive Impairment

In the Dutch famine birth cohort, famine exposure did not increase the prevalence of stroke at 41 years of age^25^ or mortality from CVD by 63 years of age.^26^ In contrast, those exposed to the Chinese great famine were more than twice as likely to have a stroke by 50 years of age compared with unexposed participants, independent of vascular risk factors.^27^ This may be because the Chinese famine was more severe and occurred in a previously undernourished population or may be due to methodological differences between studies.

Importantly, in all of these studies, the participants were relatively young (<65 years) at assessment, yet the median age for stroke is 73 years, and incidence rises with age.^28^ Further research is needed to determine whether stroke risk conferred by birthweight increases with advancing age.

Few studies have examined other prenatal exposures such as smoking, alcohol, preeclampsia and gestational diabetes, and later-life CVD. These exposures are associated with smaller brain volumes in children and adults^29^ and increased blood pressure and stroke risk in adulthood in some,^30,31^ but not all, studies.^32^ However associations have not been examined in relation to other markers of SVD.

### Small Vessel Disease

Mild variation in birthweight within the normal range, which reflects subtle variations in the intrauterine environment, predicts brain volume (reserve) and cortical configuration across the life course.^33,34^ Less is known about the effects of birth weight or prenatal malnutrition on white matter microstructure and SVD in later life. We previously reported that birth weight was positively associated with white matter integrity in the frontal lobes aged 78 years^35^ and normal appearing white matter volume,^33^ independent of hypertension, but findings are inconsistent.^33,36,37^ In community-dwelling older adults aged 59 to 81 years, lacunes, infarcts, and perivascular space severity decreased by 5% to 7% for every 100-g increase in birth weight independent of vascular risk factors, adulthood and childhood SES, education, and gestational age.^36^ This is the only study to date to examine birth weight and markers of SVD on magnetic resonance imaging other than WMH burden and brain atrophy.

Brain tissue volume in later life is a product of maximal prior brain size, inferred from intracranial volume, and tissue loss occurring with age. Birth weight positively associates with total brain volume over the age of 50 years independent of body size and vascular risk factors,^33^ and total brain volumes at the age of 68 years were smaller in famine-exposed compared with unexposed men.^37^ However, these associations are not independent of intracranial volume, suggesting nutritional deficiency or poor fetal growth disrupts brain development in early life, resulting in a smaller peak brain volume, rather than increasing vulnerability to brain tissue atrophy in later life. Intracranial volume negatively associates with risk of mild cognitive impairment, AD, and VCI^38^ and is often used as a proxy for brain reserve.^14^

## DEVELOPMENTAL ORIGINS OF HEALTH AND DISEASE, COGNITIVE IMPAIRMENT, AND DEMENTIA

Larger birth weight within the normal range is associated with better cognitive function from infancy through adulthood^39,40^ independent of parental social class.^39^ A systematic review of birth weight and later-life cognition concluded that effect sizes are small and there is insufficient adjustment for important confounders in several studies,^40^ possibly explaining inconsistent findings between small studies^40^ and larger population cohorts.^41^ In a large Swedish twin study, every 100-g decrease in birth weight increased dementia risk by 2% independent of gestational age, education, and childhood SES, and associations were not due to shared genetic or environmental factors.^41^

At 68 years of age, men, but not women, exposed to the Dutch famine had higher The Brain Age Gap Estimation (BrainAGE) scores, indicating premature brain aging.^42^ Individuals exposed to famine also performed worse on selective attention tasks at the age of 56 to 59 years^43^ and had more self-perceived cognitive problems at the age of 72 years,^44^ but this is not supported by all studies.^45^ The Chinese famine studies found prenatal famine exposure increased risk of mild cognitive impairment and dementia^46^ and resulted in poorer performance on tasks of visuomotor skills, mental flexibility and attention, and lower general cognition at the age of 51 to 56 years independent of age and education.^47^

There are several plausible biological pathways through which early-life exposures may influence children’s neurodevelopment and increase vulnerability to SVD, stroke, and dementia. These include disruption of metabolic processes underlying somatic and neural functioning and growth, altered DNA methylation, impaired white matter myelination and connectivity, fetal growth restriction, suboptimal postnatal nutrition, impaired immune defences, chronic inflammation, and neuroendocrine dysregulation.^48^

Overall, these findings suggest that the prenatal environment is an important contributor to later-life brain health. Birth weight is associated with later-life stroke and cognitive status, but whether these associations are independent of vascular risk factors is unclear. Few studies have examined associations between prenatal factors and SVD as a potential mechanistic pathway, and further studies are needed to clarify these associations.

## CHILDHOOD RISK FACTORS FOR COGNITIVE IMPAIRMENT AND DEMENTIA

Early-life IQ, education, and deprivation are determinants of cognitive aging and are associated with general health, longevity, SVD, and stroke.^49,50^

## PERINATAL BRAIN INJURY

Around 11% of the global population is born preterm^51^ (<37 weeks of gestation). For those born very preterm (<32 weeks), there is a consistent association with a magnetic resonance imaging phenotype that includes diffuse white matter disease, deep gray matter volume reduction, altered cortical configuration, and subsequent neurocognitive deficits and behavioral problems.^52^ Atypical brain structure persists into adolescence, and cognitive disadvantage programmed by preterm birth is stable into adulthood.^53^ The oldest survivors of very preterm birth are now in their fourth to fifth decades: an important area for research is to determine whether atypical brain development and cognitive disadvantage conferred by preterm birth predict increased prevalence or earlier onset of SVD and other neurodegenerative diseases.

## EARLY-LIFE COGNITIVE ABILITY

In general, cognitive ability is stable across the life course; people who score well on cognitive tests in childhood will likely score well into adulthood and old age, with correlations of 0.67 to 0.51 reported between cognition age 11 years and age 70 to 87 years.^54^

Lower early-life cognitive ability increases the risk of all-cause dementia, AD, and VCI independent of education (Table 1).^55^ In Danish conscripts (n=666 986), lower early-life intelligence was associated with higher risk of AD (hazard ratio, 1.07) and VCI (hazard ratio, 1.47) by the age of 77 years. Intrasibling and twin analyses attenuated associations, suggesting genetic and environmental factors explain some, but not all, of this association.^55^ On the contrary, whether early-life cognition influences cognitive decline is uncertain. In community samples, those with higher childhood IQ decline at a slower rate,^56,57^ but this is not supported by all studies,^54^ and it is unclear whether these associations are independent of vascular risk factors. In the 1946 British birth cohort, rates of cognitive decline were steeper in individuals with a lower intellectually enriching lifestyle.^58^ There was no association between childhood and late-life cognition in those with an intellectually enriching lifestyle, suggesting factors such as education and occupation may modify the effect of early-life IQ on cognitive decline.

## EDUCATION

Higher educational attainment is associated with better late-life cognitive functioning and reduced risk and a later onset of dementia. Meta-analysis found a 45% increased risk of dementia with low education and a 7% reduced risk for dementia for each additional year of education, although there was a wide variation of definitions of low education and inconsistent approaches to measuring education between studies.^59^

Higher levels of education predict slower rates of functional and cognitive decline in several,^60,61^ but not all,^62^ population and SVD cohorts (Table 1) independent of vascular risk factors, including hypertension, and childhood IQ. Although cognition is initially preserved in those with high education, when pathology surpasses a certain threshold, this protective mechanism may no longer be sufficient, and cognition may decline at an accelerated rate. In a 5-year longitudinal study, more years of education were associated with slower cognitive decline in those with low levels of age-related brain atrophy but faster cognitive decline in those with more atrophy.^63^ In patients with mild cognitive impairment and AD, education slows the rate of cognitive decline before diagnosis, delaying diagnosis by ≈9 years, but cognitive decline accelerates following diagnosis.^64^ The role of early-life factors in the cognitive trajectories of people with VCI is unclear and has received relatively little attention compared with AD. This is important because premorbid cognition and education are major risk factors for stroke,^50^ they predict cognitive impairment at 1 year after stroke better than more commonly included variables like stroke severity and vascular risk factors,^65,66^ and account for some differences in VCI unexplained by known risk factors.^66^

Education and SVD may have independent effects on cognition,^67^ but several studies suggest that education modifies the impact of SVD on cognition, such that those with more education are protected against SVD-related cognitive deterioration,^9^ perhaps because education teaches alternative problem-solving strategies that ameliorate the impact of SVD on executive function. This may explain inconsistent associations between vascular pathology and cognition in some individuals. Jokinen et al^61^ showed high education attenuated the association between WMH, lacunes, and cognition and predicted better cognition and slower cognitive decline, sustained functional independence, and lower mortality, independent of WMH volume and vascular risk factors, over a 7-year follow-up. However, in a community-based sample, education attenuated the effect of WMH on memory performance in those with less brain pathology, but it had the opposite effect in those with more pathology,^68^ suggesting the protective effect of education on cognition may depend on the severity, type, and stage of the brain pathology.

Educational attainment is a potentially modifiable risk factor for dementia. Raising the school-leaving age has been associated with improved later-life cognition^69^ and may partly explain the declining age-specific incident rates of dementia in many high-income countries.^13^ Furthermore, older adults who undertake further education found a measurable increase in cognitive reserve,^70^ but other studies suggest that additional education after 20 years of age has little effect on later-life cognition.^71^

In summary, higher early-life cognitive ability and more education protect against stroke and SVD independent of vascular risk factors. While these factors are also associated with better later-life cognition, associations with cognitive decline, particularly for early-life cognition, are inconsistent. Some studies suggest that higher education slows the rate of cognitive decline pre-dementia diagnosis and modifies associations between mild or moderate SVD and cognition but has the opposite effect in those with more advanced pathology, but further research, particularly in VCI, is needed to confirm these findings.

## HOW MIGHT EARLY-LIFE COGNITIVE ABILITY AND EDUCATION INFLUENCE LATER-LIFE COGNITION?

Highly educated individuals, or those with higher premorbid cognition, may have higher cognitive reserve and be able to tolerate greater neuropathological changes before clinical symptoms occur, perhaps by recruiting alternate neural networks or utilizing existing networks more efficiently. Efficient information processing speed correlates with general intelligence and is thought to rely on the structure of the white matter tracts connecting distal brain areas and myelination.^72^ Education predicts fiber tract structure in several brain areas, those with higher levels of education having more richly connected fiber tracts.^73^ More years of education is associated with specialized use of neural processing and more efficient brain networks in older adults,^74^ and white matter structure at 83 years of age correlates with age 11 IQ.^75^

Premorbid IQ and education may be a marker of brain resistance to age-related pathology such as SVD. Large-scale meta-analyses showed fewer markers of SVD with more education and higher premorbid IQ.^49^ Education and early-life cognition could reduce susceptibility to brain pathology through reducing risk factors for disease; however, associations between childhood IQ and SVD were found to be independent of vascular risk factors and adult SES,^36^ suggesting an effect of cognitive ability on brain pathology independent of adult vascular risk factors.

High levels of education or cognitive ability may be indicative of more brain reserve, evident in a larger brain or synapse count, which increase the threshold of pathological load required before the disease is clinically evident. Intelligence and brain size show a consistent modest correlation in both children and adults.^76^ Age 11 IQ and education duration predict cortical thickness in later life, and age 11 IQ accounts for over two-thirds of the cross-sectional association between cognitive ability and cortical thickness in later life.^77^

## CHILDHOOD SES

Socioeconomic disadvantage in childhood shapes neurodevelopmental and health outcomes from birth onward.^78,79^ In childhood, brain structure mediates the relationship between SES and measures of function (eg, language, attention, and memory).^80^ Children from higher SES households have access to more social and economic resources, which promote healthy development including cognitively stimulating home environments, healthier nutrition, and more stable living conditions.

Few studies have examined childhood SES and risk of stroke and SVD. In a meta-analysis, low childhood SES increases the risk of stroke (10 studies) and SVD (1 study),^49,50^ but another study did not find an association with SVD after adjustment for vascular risk factors and adult SES.^36^ Childhood SES is associated with global cognition^81,82^ in later life and may predict cognitive decline,^83,84^ although findings are inconsistent^85^ and few studies adjust for vascular risk factors (Table 1). Associations between childhood SES and late-life cognition may be mediated by educational attainment, early-life cognition, and adult SES^81,86^ as early-life cognitive ability and education are closely related to SES, both in childhood and in adulthood,^87^ but direct and independent effects of childhood SES on cognition^81,82,85^ and cognitive decline^83,84^ have been reported. SES is a complex and multidimensional construct that can be described at neighborhood or individual level. Measurement often varies between studies, and if only 1 measurement is considered, it may not always capture the full socioeconomic position of the individual.^79^ Furthermore, SES is not stable across the life course, with upward and downward mobility. Socioeconomically disadvantaged children who experience upward mobility in adulthood have better health outcomes than other disadvantaged children with static or downward mobility, suggesting upward mobility can compensate for disadvantage in childhood. Conversely, downward mobility reduces the benefits of higher SES in childhood.^82,85^ The effects of adult SES on health were also stronger for people with low compared with high childhood SES.^82^

## CONCLUSIONS

SVD is a major cause of stroke, cognitive impairment, and dementia. Current evidence suggests that favorable prenatal and early-life factors related to nutrition, SES, premorbid IQ, and education, can decrease the risk of stroke and SVD and may protect against later development cognitive decline and dementia, but more research is needed, particularly in relation to VCI. As current birth cohorts reach old age, there will be further opportunities to examine early-life factors in relation to stroke and VCI. While associations appear to be independent of vascular risk factors in several studies, inconsistencies between studies in the number and type of covariates included mean that future studies should include mediation analysis of the relationships between early-life factors, vascular risk factors, SVD, and cognition to confirm findings.

SVD increases with age, but there is little information on whether it appears at a younger age in those with adverse early-life factors. However, altered white matter diffusion measures suggesting increased vulnerability to SVD can start in young adulthood in people with high vascular risk (eg, hypertension).^88^ Future research should also examine whether early-life factors influence the age of onset of SVD. Positive early-life factors may influence health behaviors and access to socioeconomic resources beneficial to health or may increase brain integrity and resilience, reducing susceptibility of CVD.

Current evidence highlights that identifying modifiable early-life factors as targets for social policy interventions could have long-lasting impacts on health, particularly CVD and dementia. Clinicians caring for adults presenting with neurological diseases may wish to consider early-life circumstances, in addition to current risk factors, when evaluating patients. Health care providers working in pregnancy, childhood, and public health could play a vital role in informing patients and families about life-course brain health, thereby helping deliver these important policy initiatives.

## ARTICLE INFORMATION

### Sources of Funding

This study was supported by the Stroke Association/British Heart Foundation/Alzheimer’s Society “Rates Risks and Routes to Reduce Vascular Dementia” Priority Programme Award in Vascular Dementia (16VAD07); the Row Fogo Centre for Research into Ageing and the Brain (reference number AD.ROW4.35.BRO-D.FID3668413); the UK Dementia Research Institute (award numbers UKDRI–Edin002, DRIEdi17/18, and MRC MC_PC_17113), which receives its funding from DRI, Ltd, funded by the UK Medical Research Council, Alzheimer’s Society, and Alzheimer’s Research UK. J.P. Boardman works in the UK Research and Innovation Medical Research Council Centre for Reproductive Health, which is funded by the MRC Centre Grant (MRCG1002033). He holds a UKRI MRC Programme grant about early-life determinants of brain development and life course outcomes (MR/X003434/1).

### Disclosures

None.
